# Bioavailability Comparison of Nine Bioselenocompounds In Vitro and In Vivo

**DOI:** 10.3390/ijms18030506

**Published:** 2017-02-26

**Authors:** Kazuaki Takahashi, Noriyuki Suzuki, Yasumitsu Ogra

**Affiliations:** Graduate School of Pharmaceutical Sciences, Chiba University, Chiba Prefecture 263-8522, Japan; afda7638@chiba-u.jp (K.T.); n-suzuki@chiba-u.jp (N.S.)

**Keywords:** selenium, speciation, selenoamino acid, selenosugar, selenocyanate, inductively coupled plasma mass spectrometry (ICP-MS), selenoprotein P, plasma glutathione peroxidase, Caco-2

## Abstract

Selenium (Se) shows biologically ambivalent characteristics in animals. It is an essential element but becomes severely toxic when the amount ingested exceeds the adequate intake level. Its biological, nutritional, and toxicological effects are strongly dependent on its chemical form. In this study, we evaluated the toxicity and bioavailability of nine naturally occurring Se compounds, or the so-called bioselenocompounds, in vivo and in vitro. Selenite and selenocystine showed higher toxicity than the other bioselenocompounds in vitro. In an in vitro membrane permeability study using Caco-2 cells, selenomethionine and *Se*-methylselenocysteine were more efficiently transported than the other bioselenocompounds. The effect of bioselenocompounds on nutritional availability was quantitatively determined from the recovery of serum selenoproteins in Se-deficient rats by speciation analysis. In contrast to the in vitro study, there were no significant differences in the assimilation of Se into serum selenoproteins among the bioselenocompounds, including selenoamino acids, selenosugar, and inorganic Se species, such as selenite, selenate, and selenocyanate, except trimethylselenonium ion. These results indicate that animals can equally assimilate both inorganic and organic naturally occurring selenocompounds except trimethylselenonium ion, which is the urinary metabolite of excess Se. We confirmed that the bioselenocompounds except trimethylselenonium ion had equivalent nutritional availabilities.

## 1. Introduction

Selenium (Se) is an essential trace element in animals, including humans, and exists in selenoproteins as selenocysteine (SeCys). It is reported that SeCys is encoded by the UGA codon in the primary structure of selenoprotein, and that humans have 25 genes encoding selenoproteins [1]. Although humans and animals ingest Se in various chemical forms via foods and feeds, Se deficiency has been reported. Keshan disease is a disease that originated in Northeast China where the soil Se concentration is very low [2]. In clinical settings, Se deficiency is often reported in patients who receive total parental nutrition (TPN) and sick children who are given special milk for specific metabolic diseases [3]. In these cases, Se supplements are frequently prescribed.

The toxicity and bioavailability of Se strongly depend on the chemical form of Se. Se is an essential element in animals but can be very toxic when the amount ingested exceeds the nutritional intake level. In addition, the adequate physiological range between deficient and excess doses is narrow [4], being one order of magnitude (0.1–1.0 μg/g diet or mL drinking water) in experimental animals. Generally, inorganic Se species, such as selenite, are more toxic than organic Se species, such as selenomethionine (SeMet), and naturally occurring Se compounds, such as animal and plant metabolites, are less toxic than artificial Se compounds. The possible nutritional sources of Se are as follows. As plant selenometabolites, the most abundant Se species are *Se*-methylselenocysteine (MeSeCys) and its derivatives, such as γ-glutamyl-*Se*-methylselenocysteine [5]. Selenohomolanthionine (SeHLan) is also identified as a plant Se metabolite [6]. SeMet, which is the major Se metabolite in yeast, has already been used for Se supplementation [7]. As animal selenometabolites, SeCys is found in selenoproteins. Se ingested by animals within the physiological range is excreted into urine as 1β-methylseleno-*N*-acetyl-d-galactosamine (SeSug1) [8]. Beyond the physiological range, the trimethylselenonium ion (TMSe^+^) appears in urine [9]. Recently, it was reported that selenocyanate (SeCN^−^) is a Se metabolite in animal cells [10]. Other inorganic Se species, such as selenite and selenate, are also used for Se supplementation [11]. At present, there is limited information on the toxicity and bioavailability of the possible nutritional sources of Se. In addition, one of the interesting clinical issues is which chemical form of Se is the most effective and safest for use in TPN and as a food additive.

**Figure 1:**
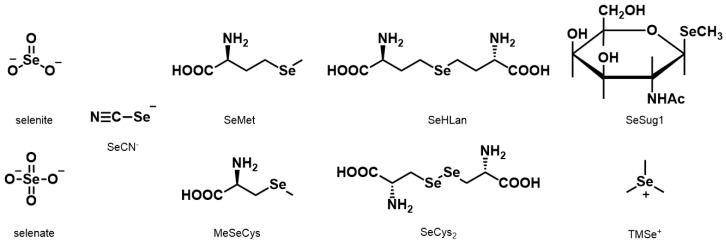
Structures of bioselenocompounds used in this study. SeCN^−^: selenocyanate, SeMet: l-selenomethionine, MeSeCys: *Se*-methylseleno-l-cysteine, SeHLan: l-selenohomolanthionine, SeCys_2_: selenocystine, SeSug1: *Se*-methylseleno-*N*-acetylgalactosamine, TMSe^+^: trimethylselenonium ion.

In this study, the nine Se compounds mentioned above were defined as bioselenocompounds, and their toxicity and bioavailability in vitro and in vivo were compared. The chemical structures of the bioselenocompounds used in this study are shown in Figure 1. In particular, to obtain quantitative data of the effects of bioselenocompounds on the recovery from Se deficiency in vivo, speciation analysis using HPLC coupled with an inductively coupled plasma mass spectrometer (ICP-MS) was adopted.

## 2. Results

### 2.1. Cytotoxicity/Proliferation Assay in Caco-2 and HepG2 Cells

Caco-2 cells were used in the in vitro permeability assay in the next experiment, and the cytotoxicity of bioselenocompounds was assayed 6 h after exposure to evaluate the harmful effects of bioselenocompounds on the cells. The cytotoxicity and/or proliferative effects of bioselenocompounds on HepG2 cells were analyzed 24 and 48 h after exposure. The cells could not be cultured for more than 6 h under the culture conditions of the in vitro permeability assay because the cells would be exposed to unexpected cellular stress.

**Figure 2:**
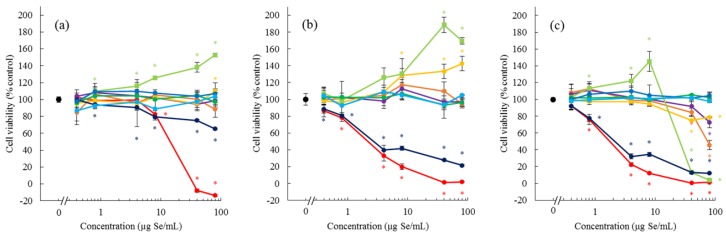
Effect of bioselenocompounds on Caco-2 and HepG2 cell viability. Caco-2 cells were exposed to selenite, selenate, ${\text{SeCN}}^{-}$, SeMet, MeSeCys, SeHLan, ${\text{SeCys}}_{2}$, SeSug1, or ${\text{TMSe}}^{+}$ for 6 h (**a**). HepG2 cells were exposed to each bioselenocompound for 24 h (**b**) and 48 h (**c**). Points and bars are means ± SD for 3 wells. Statistical analysis included the one-way analysis of variance followed by the Student’s *t*-test. The level of significance was set at *p* < 0.05, and is indicated by an asterisk (*).

Selenite significantly reduced the viability of Caco-2 cells at 8.0 μg Se/mL or higher doses (Figure 2a). SeCys_2_ also marginally but significantly reduced the cell viability at 0.8 μg Se/mL or higher doses. In contrast, MeSeCys significantly increased cell viability in a dose-dependent fashion from the dose of 0.8 μg Se/mL, indicating that MeSeCys stimulated the proliferation of Caco-2 cells. The other bioselenocompounds did not significantly change the cell viability. We also evaluated the viability of HepG2 cells for longer exposure periods (24 and 48 h) to more precisely compare the cytotoxicity of the bioselenocompounds. As was observed in Caco-2 cells, selenite and SeCys_2_ were toxic to HepG2 cells and the toxicities were dose-dependent from 0.8 and 0.4 μg Se/mL, respectively (Figure 2b,c). In addition to MeSeCys, SeMet also stimulated the proliferation of HepG2 cells exposed for 24 h, although the effect of SeMet was smaller than that of MeSeCys (Figure 2b). After 48 h exposure, MeSeCys still induced the cell proliferation at low doses; however, it showed cytotoxicity at 40 μg Se/mL or higher doses (Figure 2c). SeMet also showed marginal cytotoxicity rather than a proliferative effect. Other inorganic Se species, such as selenate and SeCN^−^, reduced cell viability at high doses, i.e., 40 and 80 μg Se/mL, respectively, after 48 h exposure.

### 2.2. Permeability Assay In Vitro

The bioselenocompounds were evaluated and compared in accordance with the standard protocol of the in vitro permeability assay using Caco-2 cells. In this assay, Caco-2 cells were exposed to the bioselenocompounds at 1.0 μg Se/mL because all of the bioselenocompounds at that dose did not show apparent cytotoxicity in the experiment mentioned above (Figure 2a).

**Figure 3:**
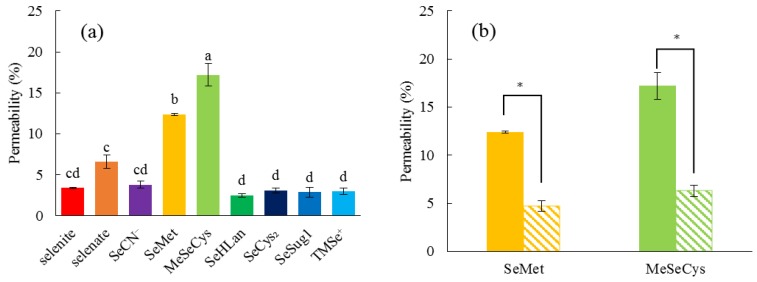
Permeability from the apical side to the basolateral side of Caco-2 cells. The permeability of the nine bioselenocompounds was compared (**a**). A 500 μL aliquot of fresh medium containing 1 μg Se/mL of each bioselenocompound was poured into the insert and the medium in the lower chamber was collected after 90 min. Values and bars are means ± SD for 3 wells. Statistical analysis was performed by the Tukey test. The level of significance was set at *p* < 0.05, and values that have a different letter are significantly different. The effects of Met on the permeability of SeMet and MeSeCys were evaluated (**b**). Hatched and solid bars represent with and without 10 mM Met, respectively. Points and bars are means ± SD for 3 wells. Statistical analysis include the one-way analysis of variance followed by the Student’s *t*-test. The level of significance was set at *p* < 0.05, and is indicated by an asterisk (*).

MeSeCys showed the highest percentage permeation (17.2% ± 1.4%) among the bioselenocompounds, followed by SeMet (12.4% ± 0.13%) (Figure 3a), i.e., these two selenoamino acids were more efficiently transported from the basolateral side to the apical side than the other species. There were no significant differences in permeability among the inorganic Se species, such as selenite (3.4% ± 0.066%), selenate (6.6% ± 0.83%), and SeCN^−^ (3.8% ± 0.44%), and among all organic Se species except MeSeCys and SeMet, such as SeHLan (2.5%± 0.23%), SeCys_2_ (3.1% ± 0.30%), SeSug1 (2.9% ± 0.60%), and TMSe^+^ (3% ± 0.42%), even though the permeability of selenate was significantly higher than those of the organic Se species. Lucifer Yellow (LY) was used to calibrate the permeability. As LY could not be transported across Caco-2 cells, its permeability would indicate non-specific transport, such as membrane leakage. LY leakage was 0.81% ± 0.36% in our experiments. Hence, selenite, SeCN^−^, SeHLan, SeSug1, and TMSe^+^ were slightly transported under our experimental conditions.

We speculated that MeSeCys and SeMet permeated the cell membrane via an amino acid transporter. Then, we evaluated whether Met would affect the permeability of MeSeCys and SeMet or not. The permeability of MeSeCys and SeMet was reduced in the presence of 10 mM Met (Figure 3b).

### 2.3. Bioavailability of Bioselenocompounds In Vivo

**Figure 4:**
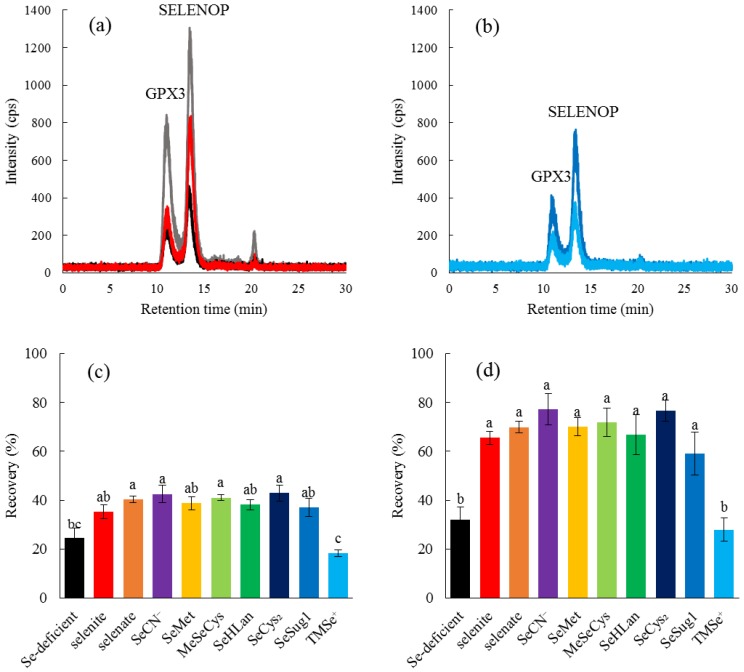
Bioavailability of nine bioselenocompounds in Se-deficient rats. Se distribution in serum was detected by LC-ICP-MS. Sera were collected from Se-adequate rats (**a**) and Se-deficient rats administered selenite (**a**), saline (**a**), or urinary selenometabolites, such as SeSug1 (**b**) or ${\text{TMSe}}^{+}$ (**b**). Se bioavailability was evaluated from the recovery of Se in two serum selenoproteins, i.e., glutathione peroxidase (GPX3) (**c**) and selenoprotein P (SELENOP) (**d**), in the sera of Se-adequate rats, Se-deficient rats administered saline, selenite, selenate, ${\text{SeCN}}^{-}$, SeMet, MeSeCys, SeHLan, ${\text{SeCys}}_{2}$, SeSug1, or ${\text{TMSe}}^{+}$. Values and bars are means ± SD for 3 wells. Statistical analysis was performed by the Tukey test. The level of significance was set at *p* < 0.05, and values that have a different letter are significantly different.

Two major serum selenoproteins were detected by LC-ICP-MS (Figure 4a). The peak detected at the retention time of 11.0 min was assigned to extracellular glutathione peroxidase (GPX3), and the peak at the retention time of 13.5 min was assigned to selenoprotein P (SELENOP) according to our previous literature [12]. The abbreviations of the selenoproteins (GPX3 and SELENOP) were adopted in accordance with the latest nomenclature [13]. We used the recovery amounts of serum selenoproteins in Se-deficient rats as the index of Se bioavailability when a selenocompound was administered to an Se-deficient rat. Indeed, feeding the Se-deficient diet to the rat for 3 weeks effectively reduced the amounts of GPX3 and SELENOP as determined by LC-ICP-MS. The recovery of both selenoproteins was observed when selenite was administered (Figure 4a). SeSug1 showed an equivalent effect to selenite on the recovery even though it is one of the major urinary selenometabolites (Figure 4b). In contrast to SeSug1, TMSe^+^, another major urinary selenometabolite, did not have any apparent effects on the recovery (Figure 4b). Sera sampled from rats administered each bioselenocompound were analyzed for the recovery of Se in the form of selenoproteins by LC-ICP-MS, and their bioavailabilities, namely, the assimilation of Se into GPX3 and SELENOP, are summarized in Figure 4c,d, respectively. Coinciding with a previous study [14], SELENOP was more efficiently recovered than GPX3 in the Se-deficient rats 24 h after the repletion of the bioselenocompounds except for TMSe^+^. There were no significant differences in the recovery of GPX3 among all the bioselenocompounds except for TMSe^+^ (Figure 4c). As SELENOP was more efficiently recovered, the recovery of SELENOP by all the bioselenocompounds except TMSe^+^ was more obvious than that of GPX3 (Figure 4d). The amounts of SELENOP in rats administered the bioselenocompounds except for TMSe^+^ were significantly different from that in the Se-deficient rats. Therefore, there were no significant differences in the bioavailability in vivo among all the bioselenocompounds except TMSe^+^. These in vivo results did not reflect the results of the in vitro assay.

## 3. Discussion

Selenite and SeCys_2_ showed higher toxicity than the other bioselenocompounds, in agreement with previous literature [15]. Selenite is more easily reduced to selenide than selenate in living organisms [16], and selenide is recognized as the ultimate toxicant of selenite and selenate [17]. Thus, the toxicity of selenite is suggested to be equivalent to that of selenide, and the high toxicity of selenite reflects its easy reduction to selenide. The high toxicity of SeCys_2_ is quite enigmatic. Generally, organic Se species, such as selenoamino acids, are less toxic than inorganic Se species. However, the high toxicity of SeCys_2_ is comparable to that of selenite although other selenoamino acids, such as SeMet, MeSeCys, and SeHLan, are actually less toxic than selenite. Although the precise mechanisms are still unclear, SeCys_2_ is expected to be reduced to SeCys in cells. It is reported that the selenyl group (–SeH) in SeCys is highly reactive compared to a sulfhydryl group (–SH) [18]. For instance, the pKa values of SeCys and Cys are 5.2 and 8.3, respectively. This indicates that the selenyl group (–SeH) in SeCys exists as a selenide (–Se^−^) at physiological pH (near 7.0), whereas the sulfhydryl group remains unchanged. In addition, the selenyl group is more sensitive to oxidation than the sulfhydryl group. Thus, the high reactivity and sensitivity to oxidation produce toxic effects in cells, resulting in the denaturation of proteins and the generation of reactive oxygen species. It is suggested that SeCys_2_ exerts its toxicity by appearing as a selenide (–Se^−^) in cells. Indeed, the severe toxicity of SeCys_2_ was observed after exposure for 24 and 48 h. This suggests that the reduction of the diselenide bond by an unknown mechanism requires a substantial amount of time for completion. Consequently, the toxicity of bioselenocompounds reflects the susceptibility of the bioselenocompounds to the production of highly reactive selenide moieties (HSe^−^ or –Se^−^).

MeSeCys induced the proliferation of Caco-2 and HepG2 cells, but exhibited cytotoxicity at a high dose and after a long exposure time. SeMet showed a weaker effect on cell proliferation than MeSeCys. No other bioselenocompounds induced the cell proliferation. MeSeCys is a promising agent in cancer prevention and treatment [19]. It is reported that *S*-methylcysteine (MeCys) exerts a chemopreventive effect [20]. Thus, the cytotoxicity of MeSeCys is due to the structure of the MeCys derivative. In contrast to the cytotoxicity, the proliferative effects of MeSeCys, MeCys, SeMet, and methionine (Met) have not yet been reported. Although Met is an essential amino acid for cell growth, our culture conditions, which are standard for Caco-2 and HepG2 cells, contain the required amount of Met. Indeed, Met and MeCys exhibited no proliferative effects (data not shown), and the other bioselenocompounds also did not show the effects (Figure 2). Hence, the proliferative effects seem to be specific to MeSeCys and SeMet. The cell proliferative effects of MeSeCys and SeMet are expected to be novel pharmacological effects in addition to the Se source. Further studies are needed to confirm their effects.

Although SeMet and MeSeCys more efficiently permeated Caco-2 cells than inorganic Se species, in agreement with the previous literature [21], other selenoamino acids, such as SeHLan and SeCys_2_, were comparable as inorganic Se species (Figure 3). The fact that the permeation of SeMet and MeSeCys was inhibited by the presence of excess Met suggested that these two selenoamino acids were transported by the same transporter as Met. In other words, both SeMet and MeSeCys are transported by an ordinary l-amino acid transporter without being distinguished from Met. As SeHLan and SeCys_2_ can be recognized as a dimeric form of amino acids on the basis of their lanthionic and disulfide bonds, respectively, these selenoamino acids are less easily transported than a monomer by the transporter. Consequently, it can be concluded from our in vitro experiments that the monomeric forms of selenoamino acids, such as MeSeCys and SeMet, are more efficiently absorbed than other Se forms.

We also evaluated the bioavailability of bioselenocompounds in in vivo experiments. Specifically, we evaluated the bioavailability from the recoveries of two serum selenoproteins, GPX3 and SELENOP, in Se-deficient rats by speciation analysis with an LC-ICP-MS. We have already established a speciation analysis for the determination of the two serum selenoproteins [12], which gives us more qualitative data than Western blotting and enzyme activity determination. In particular, the enzyme activity of SELENOP is still unknown. Hence, the bioavailability of bioselenocompounds was quantitatively determined in this study. Quantitative methods indicated no significant differences in bioavailability among the bioselenocompounds except TMSe^+^ (Figure 4), although the in vitro experiment showed that the permeability of MeSeCys and SeMet was significantly larger than that of the other Se species. The discrepancy between in vitro and in vivo results is interesting and important for the consideration of the nutritional availability of bioselenocompounds. We offer two explanations for the discrepancy. First, monomeric selenoamino acids are more rapidly absorbed in the gastrointestinal tract than other Se species. However, the monomeric selenoamino acids have a methylselenyl group (CH_3_Se–) in their molecules. To realize the assimilation of Se from a selenocompound having a methylselenyl group into selenoproteins, the demethylation step is needed. However, the molecular mechanisms underlying the demethylation reaction are unclear at present. The demethylation reaction seems to be the rate-limiting step for the recovery of selenoproteins in Se-deficient rats. Indeed, as both SeSug1 and TMSe^+^ are urinary selenometabolites, we speculate that both compounds would be less efficiently assimilated into selenoproteins in animals even though the animals are Se-deficient. However, SeSug1 is assimilated and TMSe^+^ is not. SeSug1 is monomethylated and TMSe^+^ is trimethylated; thus, a more highly methylated Se is less available as the source of selenoproteins. Even a monomethylated form of Se species could be less efficiently assimilated than a nonmethylated form. Therefore, although SeMet and MeSeCys are more rapidly absorbed than the other Se species, SeMet and MeSeCys are less efficiently assimilated into selenoproteins, i.e., the net bioavailability of SeMet and MeSeCys is comparable to that of the other Se species except the highly methylated one, TMSe^+^. Second, the bioselenocompounds are metabolized/decomposed before absorption in the gastrointestinal tract. For instance, the bioselenocompounds are metabolized in intestinal flora. Similar to the nutritional availability of Se compounds, the toxicity of the arsenic (As) compound is also dependent on its chemical species. It has been reported that As toxicity is modulated by gut microbiota [22]. The bioselenocompounds seem to be metabolized by intestinal flora to common selenometabolite(s) of microbiota, resulting in the equal absorption of the bioselenocompounds except TMSe^+^. It is reported that TMSe^+^ is not metabolized by Corynebacterium, which is widely distributed in the gut microbiota of animals [23]. Hence, the bioselenocompounds except TMSe^+^ are considered nutritionally equal when administered orally. The molecular mechanisms underlying the demethylation of methylated Se species and the effects of intestinal flora should be further investigated. It is reported that the Se content of SELENOP was independent of sex and age [24]. Only male young rats were used in this study. Hence, the in vivo evaluations using female and elderly rats are needed in our future experiments.

In addition to MeSeCys from plants, i.e., vegetables, cereals, nuts, and fruits, and SeCys in selenoproteins in animals, i.e., meat and fish, selenoneine, (2*S*)-3-(2-selenoxo-2,3-dihydro-1*H*-imidazol-4-yl)-2-(trimethylazaniumyl)propanoate (2-selenyl-*N*^α^,*N*^α^,*N*^α^-trimethyl-l-histidine), which is the major Se metabolite in marine animals, is also a possible nutritional source of Se for people who are ichthyophagous [25]. In this study, selenoneine was not used as a bioselenocompound because it could not be chemically synthesized. According to the findings of this study, selenoneine would be the most preferable source of Se because it is a monomeric amino acid and contains non-methylated Se in its molecule. These structural features are expected to result in increased absorption, metabolism, and assimilation into selenoproteins. We are trying to synthesize selenoneine in order to evaluate its nutritional value. 

Inorganic Se compounds are more economical than organic Se compounds. Thus, selenite and selenate have been used in the clinical setting even though they have higher toxicity than organic Se compounds, such as selenoamino acids. SeCN^−^ is reported to be less toxic than selenite [10]. It is assimilated into selenoproteins in a manner comparable to selenite in this study. These results indicate that SeCN^−^ is a suitable inorganic Se form that is economical and safe for Se supplementation.

## 4. Materials and Methods

### 4.1. Materials

Sodium selenate and potassium selenocyanate (SeCN^−^) were purchased from Wako Pure Chemical Industries, Ltd. (Osaka, Japan). Sodium selenite and seleno-l-methionine (SeMet) were purchased from Nacalai Tesque (Kyoto, Japan). *Se*-Methylseleno-l-cysteine (MeSeCys) and l-selenocystine (SeCys_2_) were purchased from Acros Organics (Waltham, MA, USA) and Tokyo Chemical Industry Co., Ltd. (Tokyo, Japan), respectively. Trimethylselenonium iodide (TMSe^+^) was purchased from Tri Chemical (Uenohara, Japan). l-Selenohomolanthionine (SeHLan) and *Se*-methylseleno-*N*-acetylgalactosamine (SeSug1) were synthesized in our laboratory in accordance with our previous work [6,8]. The chemical structures of the bioselenocompounds used in this study are shown in Figure 1.

### 4.2. Cell Culture and Cell Viability Assay

A human colon carcinoma cell line (Caco-2) and a human hepatocyte carcinoma cell line (HepG2) were obtained from Riken BioResource Center (Wako, Japan). The composition of the passaging medium for Caco-2 or HepG2 was Eagle’s Minimum Essential Medium (MEM) with 20% FBS, 1% non-essential amino acid (NEAA), and 1% penicillin-streptomycin, or Dulbecco’s Modified Eagle’s Medium (DMEM) with 10% FBS and 1% penicillin-streptomycin, respectively. Caco-2 cells were seeded on a 96-well plate at 5500 cells/well and incubation was carried out for 2 days. The cells were exposed to selenite, selenate, SeCN^−^, SeMet, MeSeCys, SeCys_2_, SeHLan, SeSug1, or TMSe^+^ at concentrations of 0.4, 0.8, 4, 8, 40, and 80 μg Se/mL for 6 or 24 h. Cell viability was determined by the CellTiter 96^®^ Aqueous One Solution Cell Proliferation Assay (Promega, Madison, WI, USA) based on mitochondrial respiratory activity. HepG2 cells were seeded on a 96-well plate at 5000 cells/well and incubation was carried out for 1 day. Then, the cells were exposed to a Se compound, and cell viability was determined by using the same protocol as that mentioned above.

### 4.3. In Vitro Transport of Se Compounds

Confluent Caco-2 cells at the cell density of around 20,000 cells/cm^2^ were harvested by trypsinization and suspended in basal medium (Corning, Corning, NY, USA) containing MITO+ Serum Extender (Corning) at the cell density of 4 × 10^5^ cells/mL. A 0.5 mL aliquot of the cell suspension was seeded on the inserts of a Falcon 24-Multiwell Insert System (Corning). The inserts were transferred onto a 24-well plate that contained 1 mL of basal medium, and incubation was carried out for 24 h. Then, the basal medium was carefully removed from the wells and the inserts, and 1.0 and 0.5 mL aliquots of the differentiation medium (Entero-STIM) containing MITO+ Serum Extender (Corning) were added to the wells and the inserts, respectively. The plate was incubated for another 3 days to induce the formation of a monolayer of differentiated Caco-2 cells. The medium was carefully removed from the inserts and the inserts were washed with transport buffer (HBSS + 10 mM HEPES, pH 7.4). Then, a 350 μL aliquot of each Se compound solution in transport buffer or this solution with 10 mM l-methionine (Wako) was added to the insert chamber. These inserts were transferred onto a plate and a 50 μL aliquot was withdrawn from the chamber of each insert for measurement of the initial concentration of the donor chamber. One milliliter of transfer buffer was added to the plate wells and the plate was incubated on an orbital shaker at 37 °C for 90 min in a CO_2_ incubator. The transport buffer in the lower chamber was collected to determine Se concentration 90 min after the incubation. Se concentrations were measured by an inductively coupled plasma mass spectrometer (ICP-MS, Agilent 7700cx, Agilent, Hachiouji, Japan). The Se compound solution in the chamber of each insert was removed and a 300 μL aliquot of 300 μM Lucifer Yellow (LY) solution was added to the upper chamber. Then, the concentration of LY that permeated the lower chamber was determined by a fluorescence photometer (excitation wavelength: 485 nm, emission wavelength: 538 nm). Monolayers producing 0.3%–2.0% LY flux were recognized as intact monolayers of differentiated Caco-2 cells. Monolayers with flux exceeding 2.0% were excluded from the experiment.

### 4.4. Animal Care

All animal experiments were carried out according to the Principles of Laboratory Animal Care (NIH version, revised 1996) and the Guidelines of the Animal Investigation Committee, Chiba University, Japan (28-60, 28/01/2016).

Specific pathogen free (SPF) male Wistar rats (4 weeks of age) were purchased from Japan SLC (Shizuoka, Japan) and were housed in a humidity-controlled room maintained at 25 ± 2 °C with a 12 h light-dark cycle. The rats were fed a commercial diet (MF, Oriental Yeast Co., Ltd., Tokyo, Japan) and tap water ad libitum. After a one-week acclimation period, diet and water were changed to a Se-deficient diet (Oriental Yeast) and Milli-Q water (18.3 MΩ·cm), respectively. The rat fed the Se-deficient diet and the Milli-Q water for 3 weeks was used as an Se-deficient rat. The rats continuously fed the commercial diet and tap water served as the positive control.

### 4.5. Bioavailability Assay In Vivo

The Se-deficient rats were orally administered each Se compound in saline at a dose of 10 μg Se/rat once a day for 2 consecutive days. The saline-administered rats served as the negative control. All the rats were sacrificed 24 h after administration by exsanguination under anesthesia. Non-heparinized blood was collected and clotted blood was centrifuged at 1600× *g* for 10 min to obtain serum. A 20 μL aliquot of the serum was applied to an HPLC coupled with an ICP-MS to analyze the distribution of Se. The HPLC system (Prominence, Shimadzu, Kyoto, Japan) consisted of an on-line degasser, an HPLC pump, a Rheodyne six-port injector, and a multi-mode size exclusion column (Shodex GS-520HQ, exclusion size >300,000 kDa, 7.5 i.d. × 300 mm with a guard column; Showa Denko, Tokyo, Japan). The multi-mode size exclusion column (GS-520HQ) was eluted with 50 mM Tris-HCl, pH 7.4, at the flow rate of 0.6 mL/min. The eluate was introduced directly into the ICP-MS nebulizer to detect Se at *m*/*z* 82.

### 4.6. Statistical Analysis

All determinations were carried out in three replicates, and the results are shown as means ± SD. Statistical analysis was done by applying the Student’s *t*-test or one-way analysis of variance (ANOVA) with the Tukey test. A probability of *p* < 0.05 was considered to be statistically significant.

## 5. Conclusions

Although there were significant differences in Se transport among the bioselenocompounds available in our laboratory in vitro, Se in the bioselenocompounds except TMSe^+^ was equally assimilated into serum selenoproteins in vivo. In this sense, low-toxicity inorganic Se species, such as SeCN^−^, could be a potential form for Se supplementation in addition to selenoamino acids, such as SeMet and MeSeCys.

## Abbreviations

ICP-MS	Inductively Coupled Plasma Mass Spectrometry
GPX3	Plasma glutathione peroxidase
MeSeCy	Se-Methylselenocysteine
SeCys_2_	Selenocystine
SeHLan	Selenohomolanthionine
SELENOP	Selenoprotein P
SeMet	Selenemethionine
SeSug1	1β-methylseleno-*N*-acetyl-d-galactosamine
TMSe^+^	Trimethylselenonium ion
